# Hand Lesions as a Diagnostic Clue in Unexplained SIADH

**DOI:** 10.1002/ccr3.73237

**Published:** 2026-07-27

**Authors:** Nobuko Watanabe, Takuya Omura, Takahiro Kamihara, Taiki Sugimoto, Jun Matsumoto, Tomomi Omura, Masaki Hasegawa, Mariko Shimono, Zenzo Isogai

**Affiliations:** ^1^ Department of Medical Education Hospital, National Center for Geriatrics and Gerontology Obu Japan; ^2^ Department of Metabolic Research Research Institute, National Center for Geriatrics and Gerontology Obu Japan; ^3^ Department of Endocrinology and Metabolism Hospital, National Center for Geriatrics and Gerontology Obu Japan; ^4^ Department of Cardiology Hospital, National Center for Geriatrics and Gerontology Obu Japan; ^5^ Department of Prevention and Care Science Research Institute, National Center for Geriatrics and Gerontology Obu Japan; ^6^ Department of Nephrology Nagoya University Graduate School of Medicine Nagoya Japan; ^7^ Department of Geriatric and General Internal Medicine Meitetsu Hospital Nagoya Japan; ^8^ Department of Pathology, Hospital National Center for Geriatrics and Gerontology Obu Japan; ^9^ Department of Otorhinolaryngology Center for Sensory Organ, National Center for Geriatrics and Gerontology Obu Japan; ^10^ Department of Dermatology, Hospital National Center for Geriatrics and Gerontology Obu Japan

**Keywords:** chilblains, discoid lupus erythematosus, hyponatremia, Sjögren's syndrome, syndrome of inappropriate antidiuretic hormone secretion

## Abstract

In apparently idiopathic syndrome of inappropriate antidiuretic hormone secretion (SIADH), chilblain‐like hand plaques may reveal autoimmune connective tissue disease. Skin examination, biopsy, and serology can uncover discoid lupus erythematosus with Sjögren's syndrome overlap. Serum sodium improvement after skin treatment requires caution because spontaneous stabilization cannot be excluded.

## Graphical abstract

Chilblain‐like hand lesions in a patient with otherwise unexplained SIADH prompted evaluation for autoimmune connective tissue disease. (A) Hand lesions before treatment. (B) Marked improvement after topical difluprednate therapy. (C) Skin biopsy showing basal liquefaction/interface change with superficial perivascular lymphocytic infiltration. (D) Minor salivary gland biopsy showing focal lymphocytic sialadenitis, supporting Sjögren's syndrome.

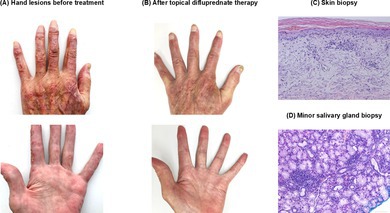

## 1 Clinical Question

In an older adult with otherwise unexplained euvolemic hypotonic hyponatremia consistent with SIADH, what diagnosis should be considered when chilblain‐like plaques are present on the dorsum of the hands, and what is the appropriate next diagnostic step?

## 2 Answer

Such lesions should prompt consideration of autoimmune connective tissue disease, including cutaneous lupus with possible Sjögren's syndrome overlap. Dermatologic evaluation with skin biopsy and autoimmune serologic testing is recommended.

## 3 Case Summary

An 82‐year‐old man presented with dizziness and fatigue. Laboratory tests showed hypotonic hyponatremia (serum sodium 119 mEq/L; serum osmolality 241 mOsm/kg) with inappropriately concentrated urine (urine osmolality 456 mOsm/kg) and elevated urinary sodium (84 mEq/L). He was euvolemic. Thyroid and adrenal function tests were normal, as were renal, hepatic, and cardiac evaluations; no diuretic use, pulmonary disease, central nervous system disease, or medication trigger was identified. Low uric acid (3.1 mg/dL) supported SIADH.

**FIGURE 1:**
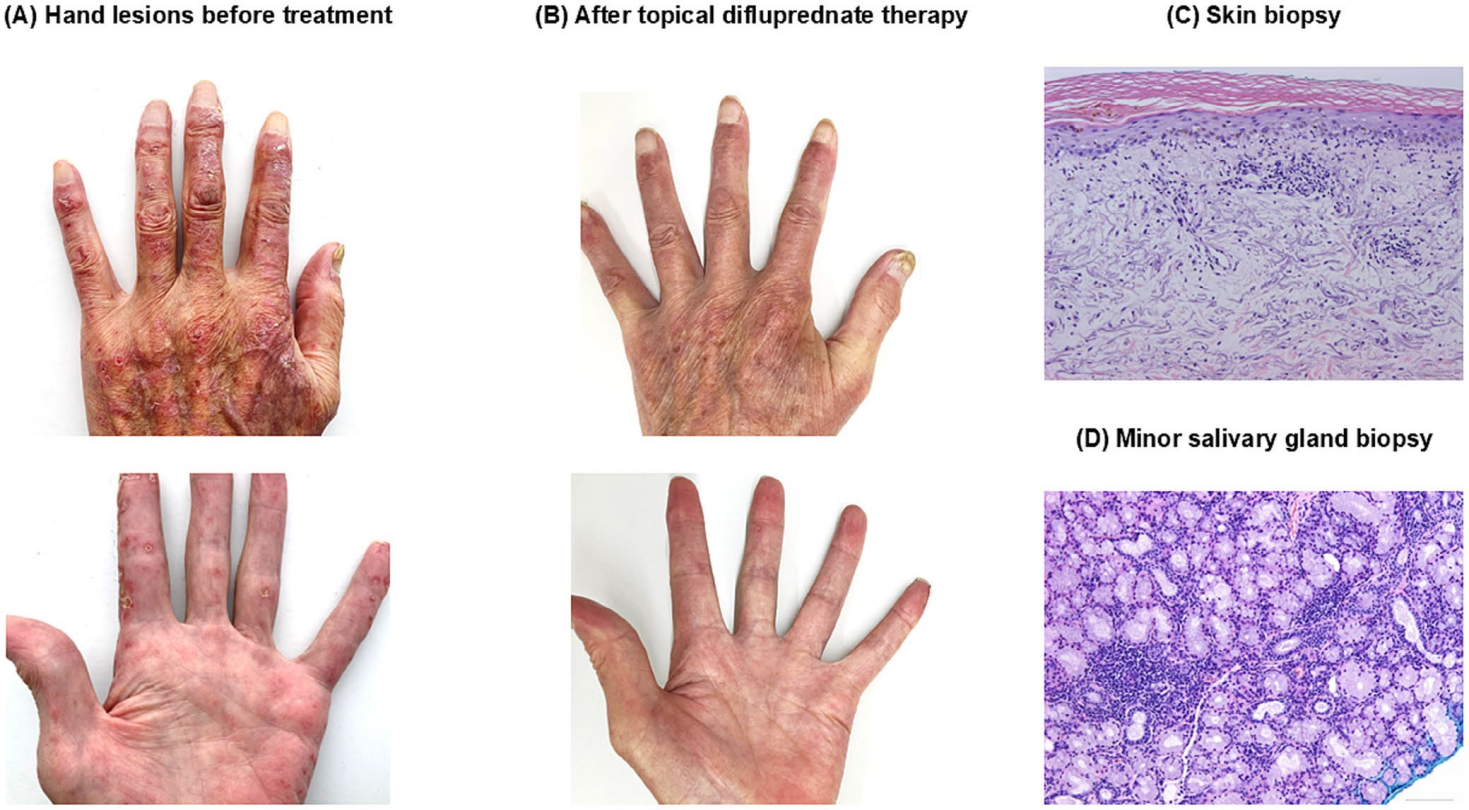
Hand lesions and biopsy findings as diagnostic clues to autoimmune connective tissue disease. (A) Hand lesions before treatment. (B) Marked improvement after topical difluprednate therapy. (C) Skin biopsy showing basal liquefaction/interface change with superficial perivascular lymphocytic infiltration. (D) Minor salivary gland biopsy showing focal lymphocytic sialadenitis, supporting Sjögren's syndrome. Scale bars in C and D = 100 μm.

Bedside inspection revealed sharply demarcated erythematous chilblain‐like plaques on the dorsum of both hands (Figure 1A). Skin biopsy supported discoid lupus erythematosus, showing basal liquefaction/interface change and superficial perivascular lymphocytic infiltration (Figure 1C). Oral dryness, high‐titer anti‐SSA/Ro and anti‐SSB/La antibodies, and minor salivary gland biopsy showing focal lymphocytic sialadenitis (Figure 1D) supported Sjögren's syndrome. There were no systemic lupus manifestations.

Fluid restriction was poorly tolerated because of thirst. Topical difluprednate improved the skin lesions (Figure 1B). Over the same period, serum sodium gradually increased from 119 mEq/L to 129 mEq/L over approximately 2 weeks and later stabilized in the 130 mEq/L range.

Inflammatory cytokines, notably interleukin‐6, may drive non‐osmotic vasopressin secretion in inflammatory conditions [1], and hyponatremia has been associated with systemic lupus erythematosus activity [2]. Sjögren's syndrome‐associated SIADH has also been reported [3]. The coexistence of discoid lupus erythematosus and Sjögren's syndrome in this patient suggests an autoimmune overlap phenotype. Topical difluprednate has limited systemic effects, and spontaneous SIADH stabilization cannot be excluded; the temporal course alone is insufficient to establish causality.

## Author Contributions

**Nobuko Watanabe:** data curation, writing – original draft. **Takuya Omura:** conceptualization, data curation, funding acquisition, methodology, visualization, writing – original draft, writing – review and editing. **Takahiro Kamihara:** data curation, supervision, writing – original draft. **Taiki Sugimoto:** data curation, supervision. **Jun Matsumoto:** data curation, supervision. **Tomomi Omura:** project administration, supervision. **Mariko Shimono:** data curation, supervision. **Zenzo Isogai:** data curation, supervision. **Masaki Hasegawa:** investigation, resources, writing – review and editing.

## Funding

This work was supported by National Center for Geriatrics and Gerontology (Grant 24‐1) and Japan Society for the Promotion of Science (Grant JP23K16812).

## Ethics Statement

This study was conducted in accordance with the Declaration of Helsinki with due consideration given to the patient. Ethical approval was not required for this single‐patient case report in accordance with institutional policy at the National Center for Geriatrics and Gerontology (NCGG).

## Consent

Written informed consent for publication was obtained from the patient.

## Conflicts of Interest

The authors declare no conflicts of interest.

## Data Availability

The data supporting the findings of this case report are available from the corresponding author upon reasonable request, subject to patient privacy and ethical restrictions.
